# Development of a novel simian adenovirus 24 based vaccine vector

**DOI:** 10.1186/1742-4690-9-S2-P309

**Published:** 2012-09-13

**Authors:** P Abbink, RR Bradley, F Ball, D Ng'ang'a, E Borducchi, DH Barouch

**Affiliations:** 1 Beth Israel Deaconess Medical Center, Boston, MA, USA

## Background

Human adenovirus serotype 5 is a potent vaccine vector, but its use has been hampered by high seroprevalence amongst people in sub-Sahara Africa. Novel adenoviral vaccine vectors from strains with lower seroprevalence worldwide are being developed that can evade pre-existing immunity. Here we describe the development of a simian Ad24 (sAd24)-based vaccine vector.

## Methods

Neutralizing antibodies against sAd24 were determined using a panel of 106 rhesus macaque sera and 128 human sera from Rwanda and South Africa using a luciferase-based adenovirus neutralization assay.

The immunogenicity of a single dose of 10E7, 10E8 or 10E9 virus particles of sAd24-SIV Gag based vector was determined in C57BL/6 mice. SIV-Gag-specific immune responses were assessed by Db/AL11 tetramer binding assays, IFN-γ ELISPOT assays and ICS assays.

## Results

Neutralizing antibodies were found in 7% of monkeys, all with titers <200. In humans from sub-Saharan Africa, 45% was positive for sAd24 neutralizing antibodies, but titers remained low and 90% had titers <200. In comparison, seroprevalence of Ad5 in sub-Saharan Africa is 86.4-89.5% with 61.1-78.7% of this population showing titers >200 and 25.1-46.8% showing titers >1000. Gag specific cellular immune responses elicited by sAd24-SIV Gag in mice are comparable to those seen with the human Ad26 and Ad28 vectors currently in development.

## Conclusion

These data suggest that sAd24 is promising for further studies as a candidate vaccine vector.

